# Sustained Remission of Lupus Panniculitis Treated With Hydroxychloroquine in a Patient With Crohn’s Disease: A Case Report

**DOI:** 10.7759/cureus.10455

**Published:** 2020-09-14

**Authors:** David D Zheng, Jazila Mantis, Dawa O Gurung, Adriana Abrudescu

**Affiliations:** 1 Internal Medicine, Icahn School of Medicine at Mount Sinai, NYC Health + Hospitals/Queens, New York City, USA; 2 Infectious Disease, Icahn School of Medicine at Mount Sinai, NYC Health + Hospitals/Queens, New York City, USA; 3 Rheumatology, Icahn School of Medicine at Mount Sinai, NYC Health + Hospitals/Queens, New York City, USA

**Keywords:** extraintestinal manifestations in inflammatory bowel disease, inflammatory bowel disease, crohn's disease, cutaneous manifestations of inflammatory bowel disease, lupus panniculitis, hydroxychloroquine

## Abstract

Extraintestinal manifestations (EIM) in inflammatory bowel disease (IBD) are common including cutaneous manifestations that either precede or follow manifestations of IBD. Cutaneous manifestations of IBD include erythema nodosum, pyoderma gangrenosum, oral lesions, and Sweet’s syndrome. Cutaneous manifestations of IBD tend to recur and extensive cases may require maintenance management with immunomodulators or biologics. However, the complications and adverse effects of long-term therapy with immunosuppressive agents are numerous and need to be considered before their initiation. We report a case of a Crohn’s disease patient with recurrent and debilitating cutaneous manifestation of lupus panniculitis that had sustained remission with hydroxychloroquine.

## Introduction

Both Crohn’s disease (CD) and ulcerative colitis (UC) are systemic disorders that often affect other organs in addition to the gastrointestinal tract. Extraintestinal manifestations (EIM) associated with inflammatory bowel disease (IBD) are common, occurring in up to 40% of patients with IBD [1]. The extraintestinal organs most commonly affected by IBD include the skin, joints, oral cavity, eyes, bone, kidneys, lungs, and biliary tract [2]. Cutaneous manifestations of IBD include erythema nodosum, pyoderma gangrenosum, oral lesions, and Sweet’s syndrome. Panniculitis is a condition in which the primary site of inflammation is in the subcutaneous adipose tissue. A large variety of subtypes of panniculitis exists including etiologies related to inflammatory processes, trauma, malignancy, or enzymatic destruction. Lupus panniculitis is an uncommon form of panniculitis with a female predominance typically presenting as erythematous nodules that is most commonly found in the proximal extremities, trunk, face, and scalp [3]. The histopathology of lupus panniculitis shows a lobular lymphocytic panniculitis with hyaline degeneration of fat in the lower dermis and subcutaneous tissue [4].

## Case presentation

**Figure 1:**
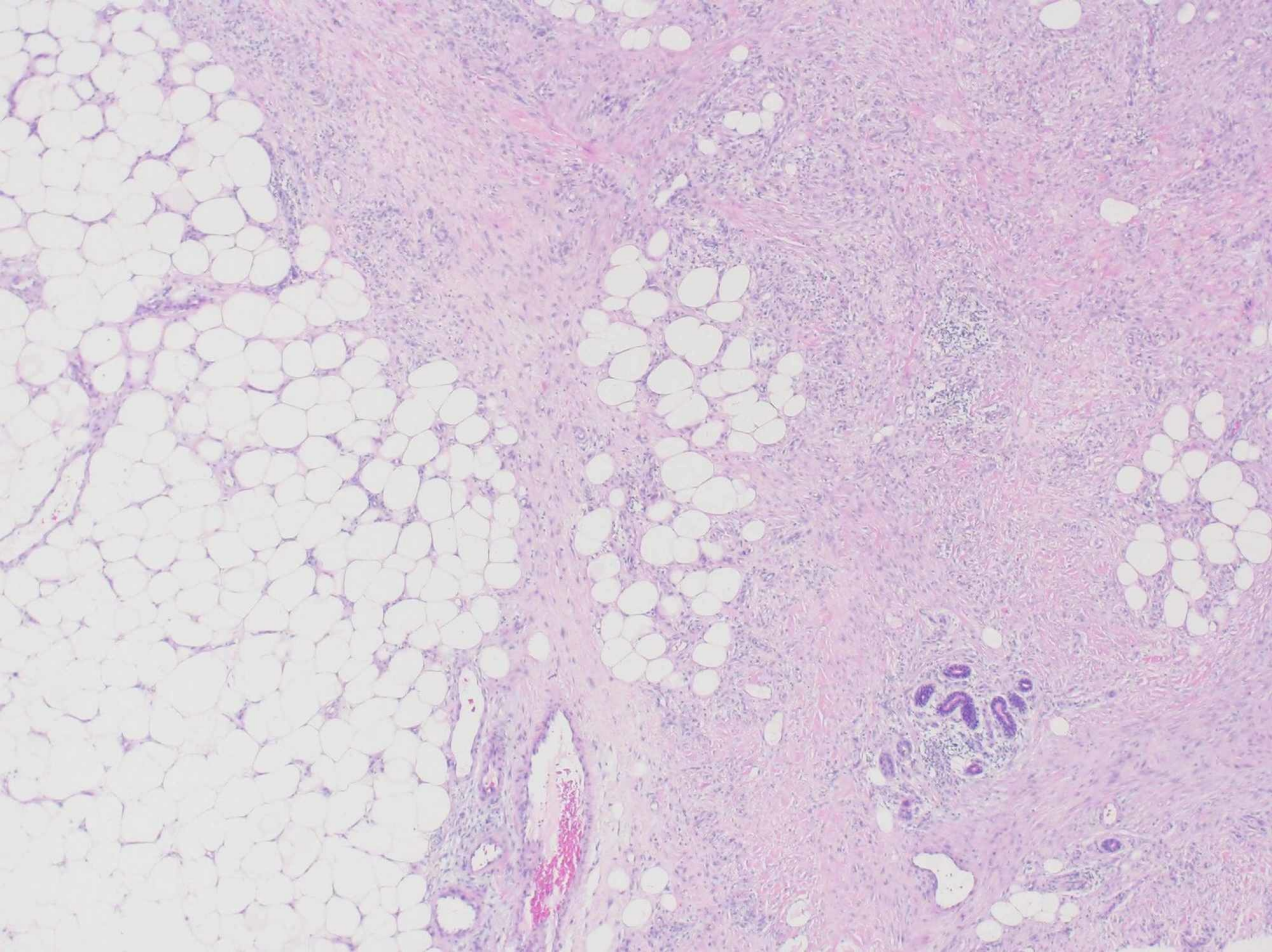
Lupus panniculitis with fat necrosis and fibrosis

A 46-year-old black female presented with multiple painful skin ulcers that had developed over a one-month period. Her medical history included bipolar disorder, migraine headache, iron deficiency anemia, and episodic watery diarrhea. Physical examination showed multiple deep ulcers with irregular borders, surrounding erythema, induration, and purulent material. Five ulcers were on her right thigh, and two on her right calf. Erythematous nodules on her left leg and abdomen were also noted which the patient reported would progress to ulcers. She was previously treated with multiple courses of antibiotics with no benefit. Laboratory examination showed elevated WBC (White blood cell) 12.6 K/μL (reference range: 3.8-10.5 K/μL), CRP (C-reactive protein) 76 mg/L (reference range: ≤5.0 mg/L), ESR (Erythrocyte sedimentation rate) 98 mm/hr (reference range: 0-20 mm/hr), positive ANA (Anti-nuclear antibodies) 1:2560 (reference range <1:80), anti-dsDNA (Double stranded DNA) antibody 104 IU/mL (reference range ≤30 IU/mL), ASCA Ab (anti-Saccharomyces cerevisiae antibodies) IgA 43 U (reference range: ≤20 U), and ASCA Ab IgG 65 U (reference range: ≤20 U). The colonoscopy showed multiple colonic ulcers, discontinuous inflammation, and crypt distortion findings supportive of Crohn’s disease. Dermatopathology of skin biopsy revealed inflammation, fibrosis, and fat necrosis consistent with lupus panniculitis (Figure 1). She did not meet diagnostic criteria for systemic lupus erythematosus. The patient was started on a tapered course of systemic steroids for Crohn’s disease and hydroxychloroquine for her debilitating lupus panniculitis with resolution of all skin lesions. A follow up colonoscopy two months later revealed colonic mucosal healing and the patient was later maintained on mesalamine for her Crohn’s disease. An attempt to discontinue hydroxychloroquine one year later resulted in recurrence of cutaneous manifestations accompanied by polyarthralgias with subsequent resolution upon resumption of hydroxychloroquine. 

## Discussion

Extraintestinal manifestations usually parallel the disease activity of IBD, but the course of some EIMs such as ankylosing spondylitis, primary sclerosing cholangitis, and pyoderma gangrenosum can be independent of intestinal inflammation [5]. Mild cutaneous manifestations of IBD might respond to local therapy with topical corticosteroids and calcineurin inhibitors [6]. However, more extensive and recurrent cutaneous manifestations of IBD stemming from severe underlying IBD require systemic immunosuppression with systemic steroids, methotrexate, azathioprine, 6-mercaptopurine and/or anti-TNF-α (Tumor necrosis factor alpha) agents [7-10]. Cutaneous manifestations have a tendency to recur and may require maintenance therapy. Side effects and complications of long-term therapy with immunosuppressive agents are numerous and need to be considered before selecting the appropriate therapy. Methotrexate, for example, can result in pulmonary toxicity and myelosuppression [11,12]. Azathioprine and 6-mercaptopurine have been shown to cause hepatotoxicity, myelotoxicity, pancreatitis, and lymphoma [13]. TNF-α inhibitors have been reported to cause neutropenia, infections (tuberculosis, bacterial infections, opportunistic infections, zoster, reactivation of hepatitis B), psoriatic skin lesions, granulomatous disease, and drug-induced lupus [14-17]. The use of hydroxychloroquine has not been well described as a treatment option for EIM in IBD. Hydroxychloroquine is also safe for use during pregnancy and lactation, which can benefit young female patients as female patients tend to have a higher prevalence of IBD [18]. Our patient with Crohn’s disease was successfully treated with hydroxychloroquine for her debilitating and recurring cutaneous manifestations. She remains symptom free on mesalamine and hydroxychloroquine three years later.

## Conclusions

Recurrent cutaneous manifestations of IBD often result from severe underlying IBD and may require systemic immunosuppression for maintenance therapy. Immunomodulators and biologics can cause many adverse effects including, but not limited to, pulmonary toxicity, neutropenia, hepatotoxicity, infections, and myelotoxicity. Hydroxychloroquine should be considered as a treatment option for cutaneous manifestations in IBD given its safety profile when compared to those of immunomodulators and biologics.
